# Supplementary material for: Calcium oscillations coordinate feather mesenchymal cell movement by SHH dependent modulation of gap junction networks
Source: Nat Commun. 2018 Dec 18;9:5377. doi: 10.1038/s41467-018-07661-5 (PMC6299091; doi:10.1038/s41467-018-07661-5)
Supplement: Supplementary file 1 — Supplementary Information [file 41467_2018_7661_MOESM1_ESM.docx]

Calcium oscillations coordinate feather mesenchymal cell movement by SHH dependent modulation of gap junction networks

Li et al.


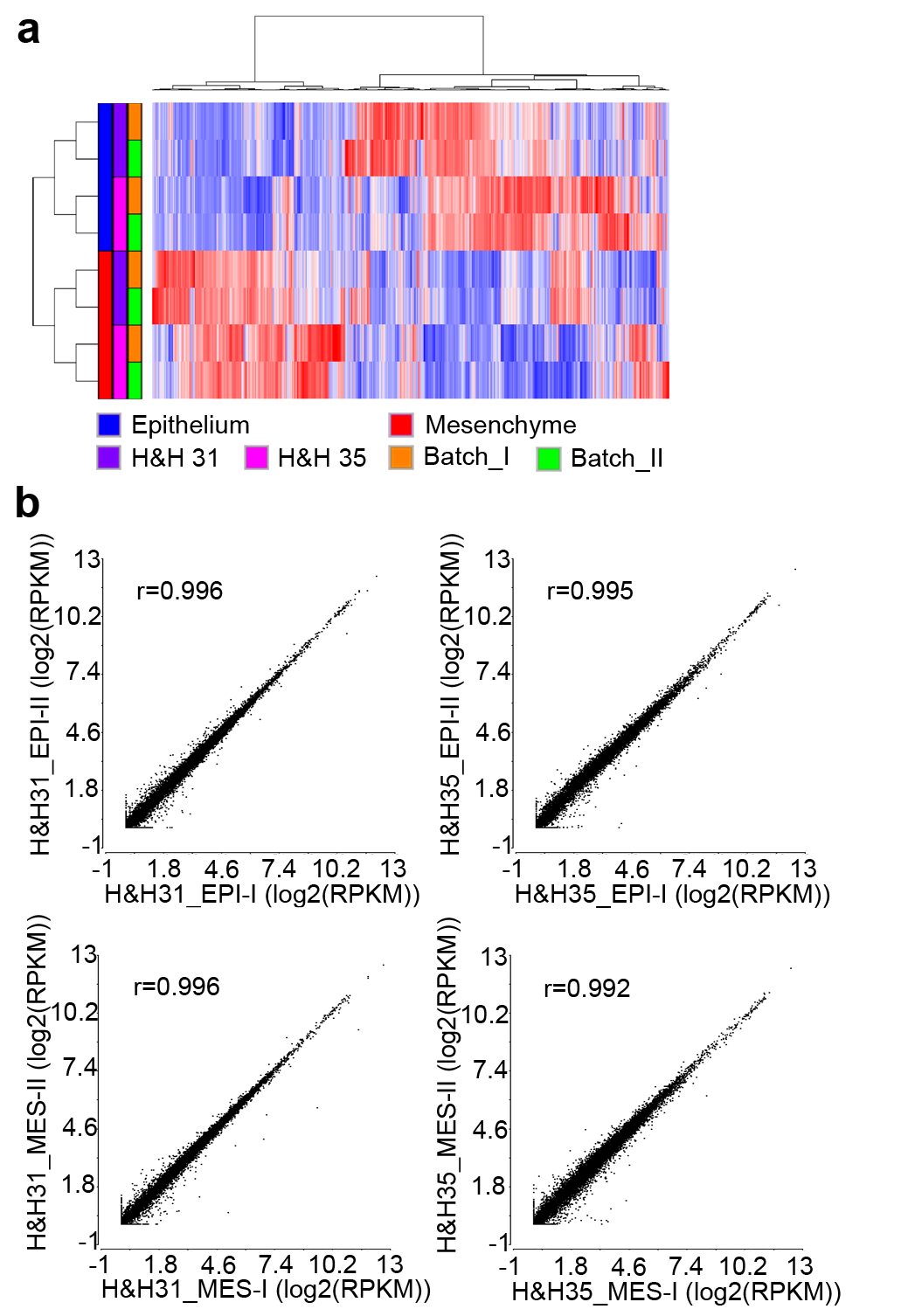


**Supplementary Figure 1 |** Transcriptome analysis of epithelium and mesenchyme tissue from H&H 31 and H&H 35 chicken embryos. (**a**) Hierarchical clustering of samples and genes. Different types of tissues show the most distinct gene expression profiles, followed by developmental stages and biological replicates (batches). (**b**) Scatter plots comparing gene expression over batches. The high linear correlation coefficient indicates high reproducibility between different batches.

**
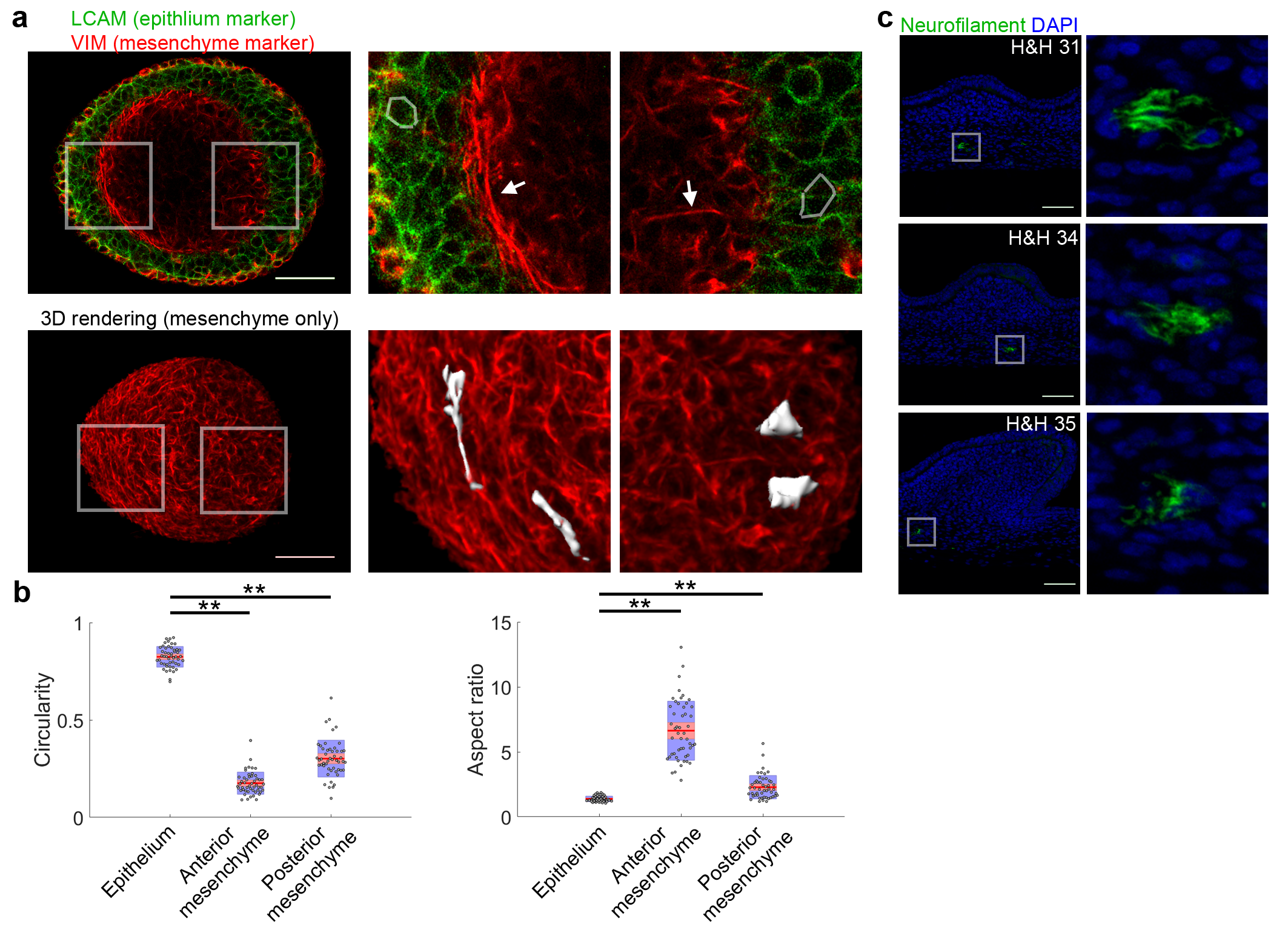
**

**Supplementary Figure 2 |** Feather bud epithelial and mesenchymal cells have distinct morphologies and molecular identities. (**a**) Upper panel: confocal (XY plane) image of H&H 34 feather bud stained with LCAM and VIM antibodies to reveal the distinct morphologies of epithelial and mesenchymal tissue. The VIM positive cells in epithelium are melanocytes and periderm cells. The epithelial tissue is mainly composed of cuboidal cells arranged in a honeycomb pattern (highlighted by white lines). The cells always tightly contact their neighbors. Mesenchymal cell boundaries are not fully occupied by the neighbors. They usually develop long filopodia (arrows). Lower panel: 3D rendering of feather mesenchyme. Anterior mesenchyme has lots of elongated, bipolar cells aligned along the epithelial-mesenchymal boundary. Posterior mesenchymal cells are mainly multipolar. White objects are surface renderings of individual mesenchymal cells. Enlarged regions are highlighted by rectangles. Scale bar, 50 μm. (**b**) Circularity and aspect ratio for feather epithelial and mesenchymal cells at different regions (n = 50). For circularity, 0 represents an infinitely elongated polygon, 1 represents a perfect circle. Customized boxplot: Mean (red) ± s.d. (pink), 95% confidence interval (violet). Dots denote individual data points. ** *P* < 0.01 (Wilcoxon Rank Test). (**c**) Neural filaments are very sparse and only exist in mesenchyme underneath feathers. Boxed regions are enlarged on the right. Scale bars, 50 μm.


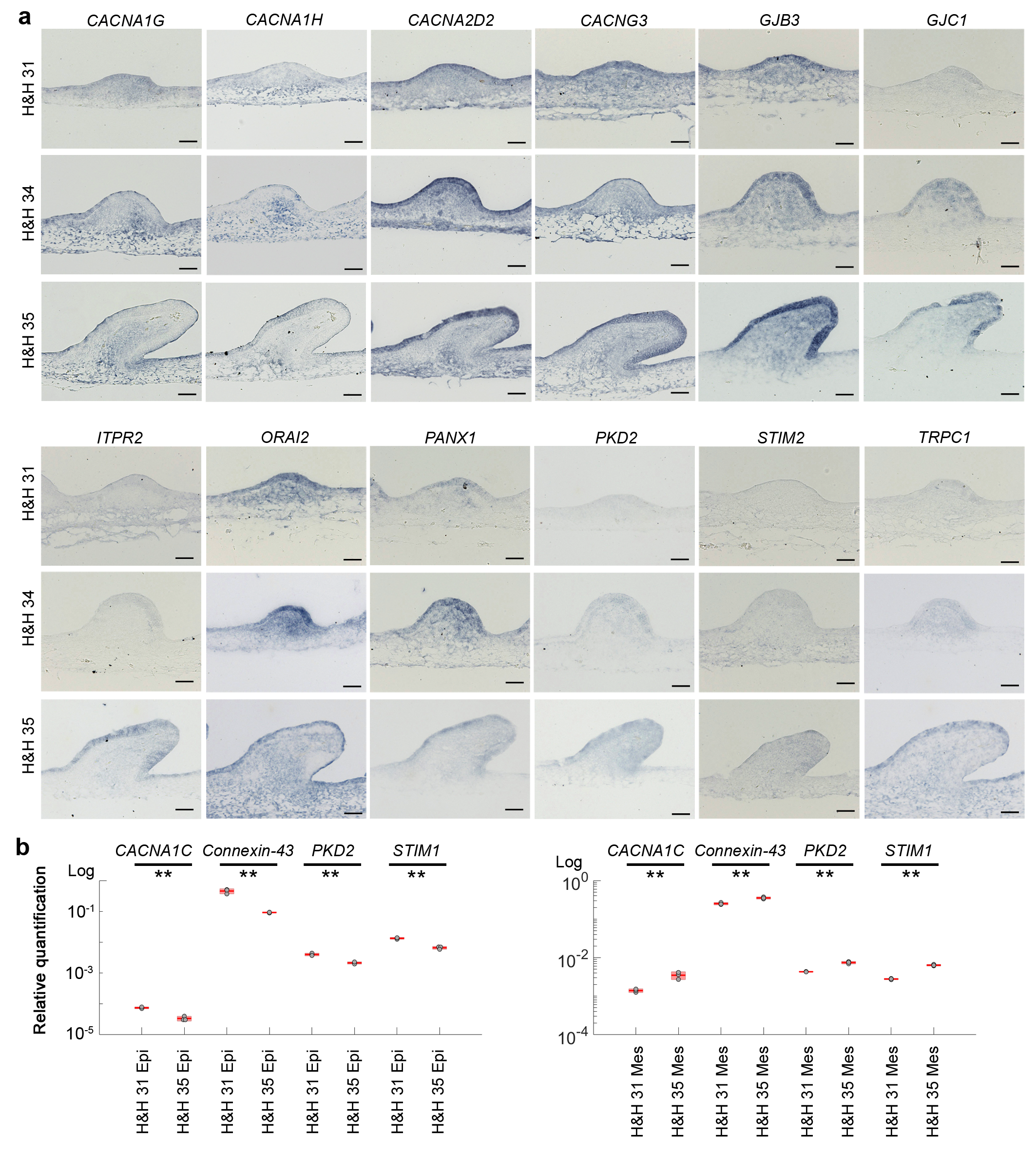


**Supplementary Figure 3 |** *In situ* hybridization and qPCR for candidate ion channel genes. (**a**) *In situ* hybridization results of other potentially interesting genes encoding ion channel components, including: 1. VGCCs: *CACNA1G* (T-type), *CACNA1H* (T-type), *CACNA2D2* (auxiliary subunit), *CACNG3* (auxiliary subunit). 2. Gap junctions: *GJB3*, *GJC1*, *PANX1*. 3. Inositol 1,4,5-trisphosphate receptor (trigger endoplasmic reticulum Ca^2+^ release): *ITPR2*. 4. CRAC channels: *ORAI2*, *STIM2*. 5. Transient receptor potential channels: *PKD2*, *TRPC1*. *CACNA1G* does not exhibit stage-dependent variations of expression according to RNA-Seq results, but its expression levels in feather mesenchyme are constantly higher than *CACNA1C* and *CACNA1H*. The *in situ* hybridization results for *ITPR1*, *PANX2,* and *TRPV2* were not shown because signals were barely detectable. Scale bar, 50 μm. (**b**) qPCR of our major candidate ion channel genes in epithelium and mesenchyme of H&H 31 and H&H 35 skins, respectively. Customized boxplot: Mean (red) ± s.d. (pink), 95% confidence interval (violet). Dots denote individual data points. ** *P* < 0.01 (n = 3, two-sample Student’s t test).


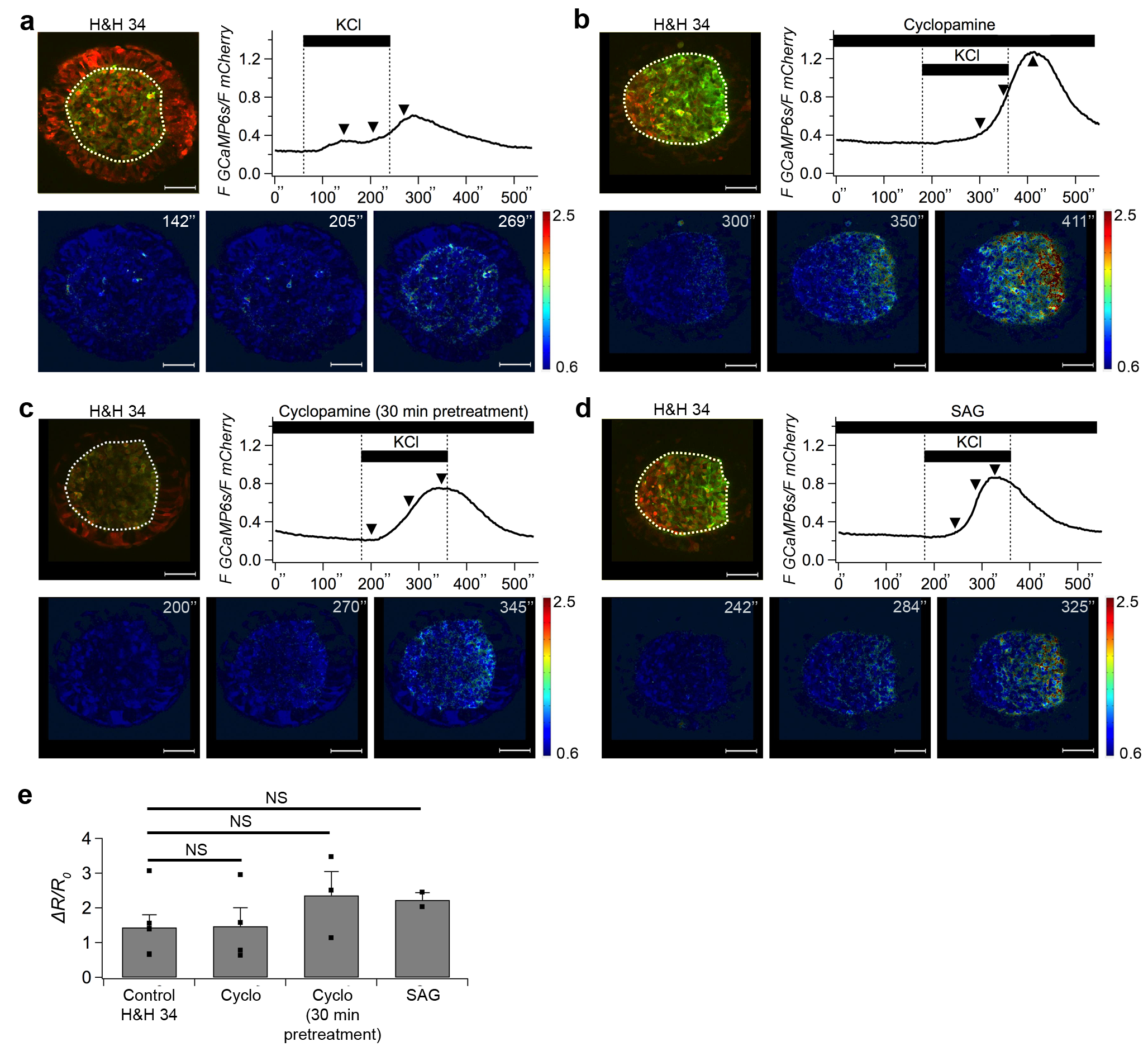
**Supplementary Figure 4 |** Short-term modulation of SHH signaling has no significant impact on feather mesenchyme Ca^2+^ responses. (**a**) Time-lapse ratiometric Ca^2+^ imaging of feather buds in H&H 34 skin explants. Feather mesenchyme, rather than epithelium, exhibited Ca^2+^ elevation after the perfusion of 100 mM KCl solution (n = 6/6). Delayed response is partially due to the bath perfusion configuration. The ROI in the mesenchyme (dotted line) was used to quantify the ratios of GCaMP6s to mCherry fluorescence intensity (’’ denotes seconds). Pseudocolor ratiometric images were generated using intensity modulated display mode, and selected at three different time points (arrowheads) in the line plot. Scale bar, 50 μm. (**b**,**c**) Pretreatment of skins for 3 min (b, n = 4/4) or 30 min (c, n = 3/3) with 5 µM Cyclopamine did not significantly decrease KCl responses. (**d**) 1 µM SAG pretreatment for 3 min did not significantly elevate the KCl responses (n = 2/2). (**e**) Quantification of Ca^2+^ changes in different treatments. *R_o_* is the baseline ratio before KCl application. *∆R* is the difference between the ratio at the peak response and *R_o_*. Dots denote individual data points. NS, not significant (Wilcoxon Rank Test).


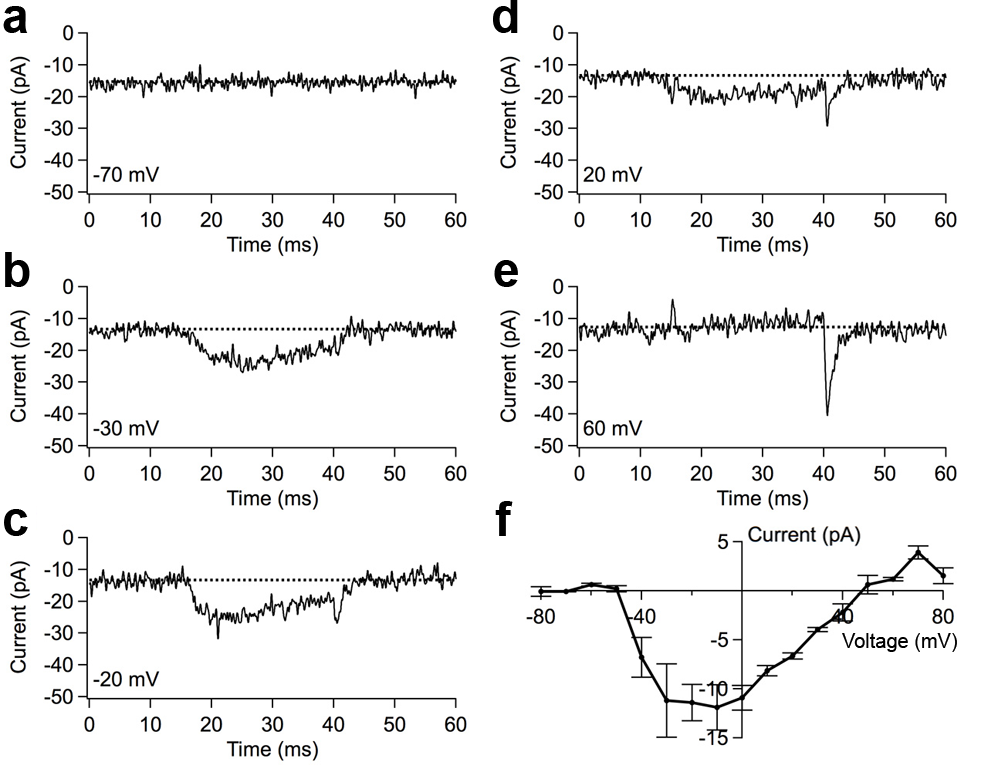


**Supplementary Figure 5** | Voltage-gated Ca^2+^ channels expressed in dissociated H&H 35 skin mesenchymal cells. (**a**-**e**) Ca^2+^ currents in response to 25 ms depolarizations. The holding potential was -80 mV, and a 10 mV step voltage increment was applied to a single mesenchymal cell. The representative inward Ca^2+^ currents were displayed at -70 mV, -30 mV, -20 mV, 20 mV and 60 mV test voltages. Each current trace was digitally filtered to reduce the noise level. Internal solution containing 10 mM TEA and 120 mM Cs-Glutamate was used to block K^+^ currents. (**f**) Peak inward currents around 20 ms were chosen to plot the current-voltage (*I-V*) curve. The *I-V* plot was not adjusted for ~11 mV liquid junction potential occurring between the internal and external solution. 3 out of 8 cells showed Ca^2+^ currents, indicating that the mesenchymal cells were composed of heterogeneous cell populations. Mean ± s.e.m.

**
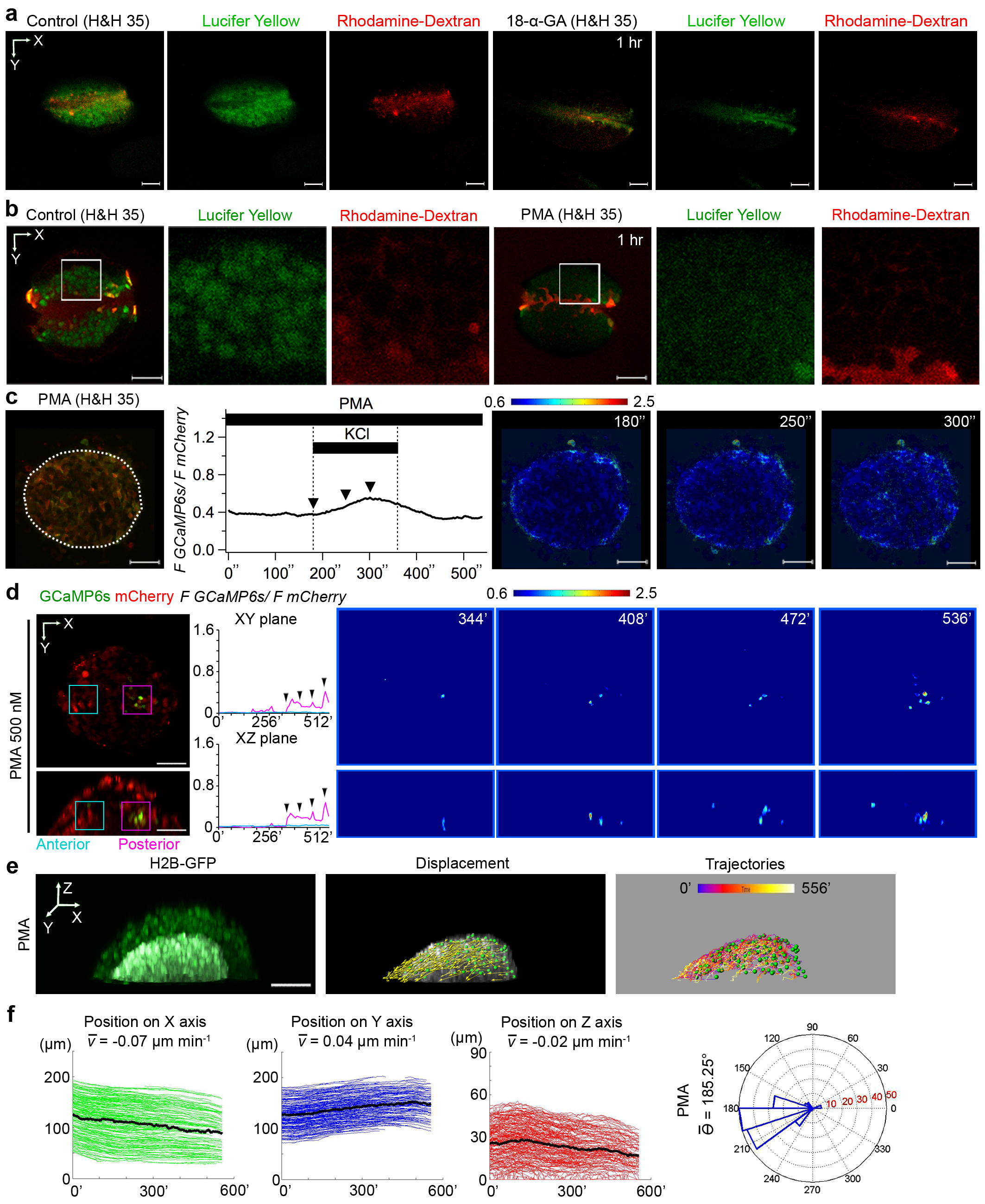
Supplementary Figure 6 |** Blocking gap junction based cell-cell communications disrupted Ca^2+^ oscillation and cell migration patterns. (**a,b**) A scrape-loading dye transfer assay demonstrated that the transfer of Lucifer yellow in H&H 35 feather mesenchyme was significantly inhibited after 1 hr pretreatment with 100 μM 18-α-GA (n = 4/4) or 500 nM PMA (n = 6/10). The high molecular weight Rhodamine-Dextran cannot pass through gap junction channels and hence largely stayed at the scrape site. Scale bars, 50 μm. (**c**) Pretreatment of H&H 35 skin with 500 nM PMA for 30 min significantly reduced the KCl induced Ca^2+^ influx in feathers (n = 5/5). (**d**) Treating skins with 500 nM PMA dramatically reduced the number of cells with high Ca^2+^ levels during feather elongation, although some mosaic Ca^2+^ transients were still observed. Quantification of *F GCaMP6s/F mCherry* was conducted for the anterior (cyan rectangle) and posterior (magenta rectangle) mesenchyme and demonstrated in line plot. Pseudocolor images of *F GCaMP6s/F mCherry* are shown at selected time points (arrowheads). Scale bar, 50 μm. (**e**) 4D cell nucleus imaging of a feather bud treated with 500 nM PMA. Feather mesenchyme is highlighted in white. Scale bar, 50 μm. (**f**) Plotting mesenchymal cell positions along the X, Y, Z axes over time. The cells mainly moved in an upward direction (n = 426).


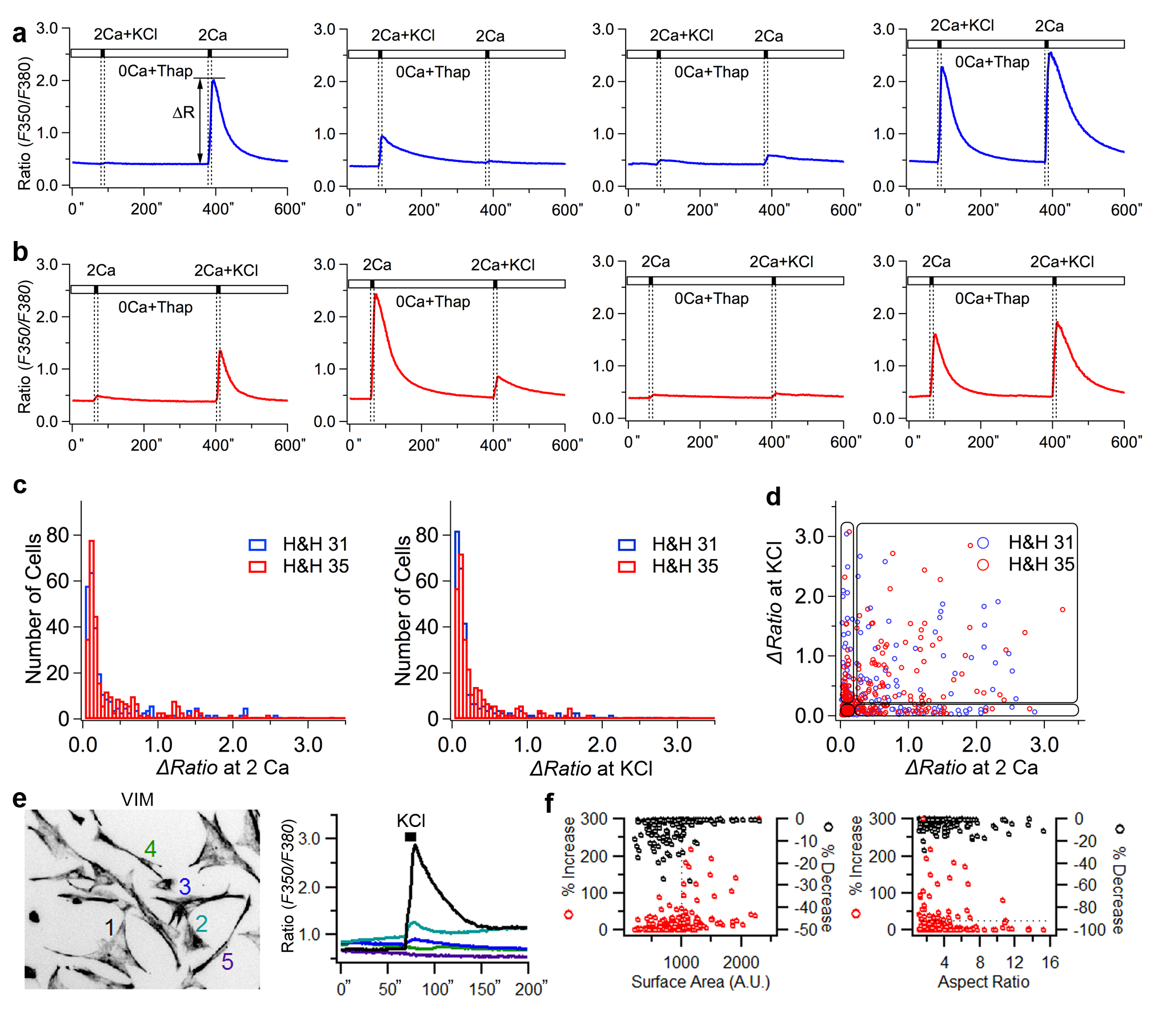


**Supplementary Figure 7 |** Heterogeneous subpopulations of mesenchymal cells with distinct channel profiles. (**a**) Cytosolic free Ca^2+^ levels in single H&H 31 mesenchymal cells were measured using AM-Fura-2 dyes. The ratios of Fura-2 fluorescence emission intensity at excitation wavelength of 350 nm (*F350*) to *F380* were calculated as readouts of change in Ca^2+^ levels. ” in x-axis denotes seconds. To deplete the ER Ca^2+^ store, cells were incubated in 0 mM Ca^2+^ solution including 5 µM Thapsigargin for 5 to 10 min before recordings. Sequential application of 100 mM KCl solution (including 2 mM Ca^2+^) and 2 mM Ca^2+^ solution without KCl was used to distinguish cells expressing VGCCs and/or CRAC channels (n = 293). The application sequence of the two solutions was randomly chosen throughout the recordings. (**b**) Dissociated mesenchymal cells from H&H 35 skins also had heterogeneous response patterns (n = 312). (**c**) Quantification of cell response strength to Ca^2+^ alone or KCl solution. *∆Ratio* is the change in ratio between the peak response ratio and baseline ratio. (**d**) Scatter plot indicates highly heterogeneous expression and/or activity of VGCCs and CRAC channels in mesenchymal cells. (**e**) Mesenchymal cells were identified by vimentin staining after Ca^2+^ measurements. Fast application of KCl solution for 10 s induced immediate Ca^2+^ responses. Five cells labeled as colored numbers were chosen to plot the ratios. (**f**) Scatter plots of Ca^2+^ responses vs. surface area (n = 151) or aspect ratio (n = 151).


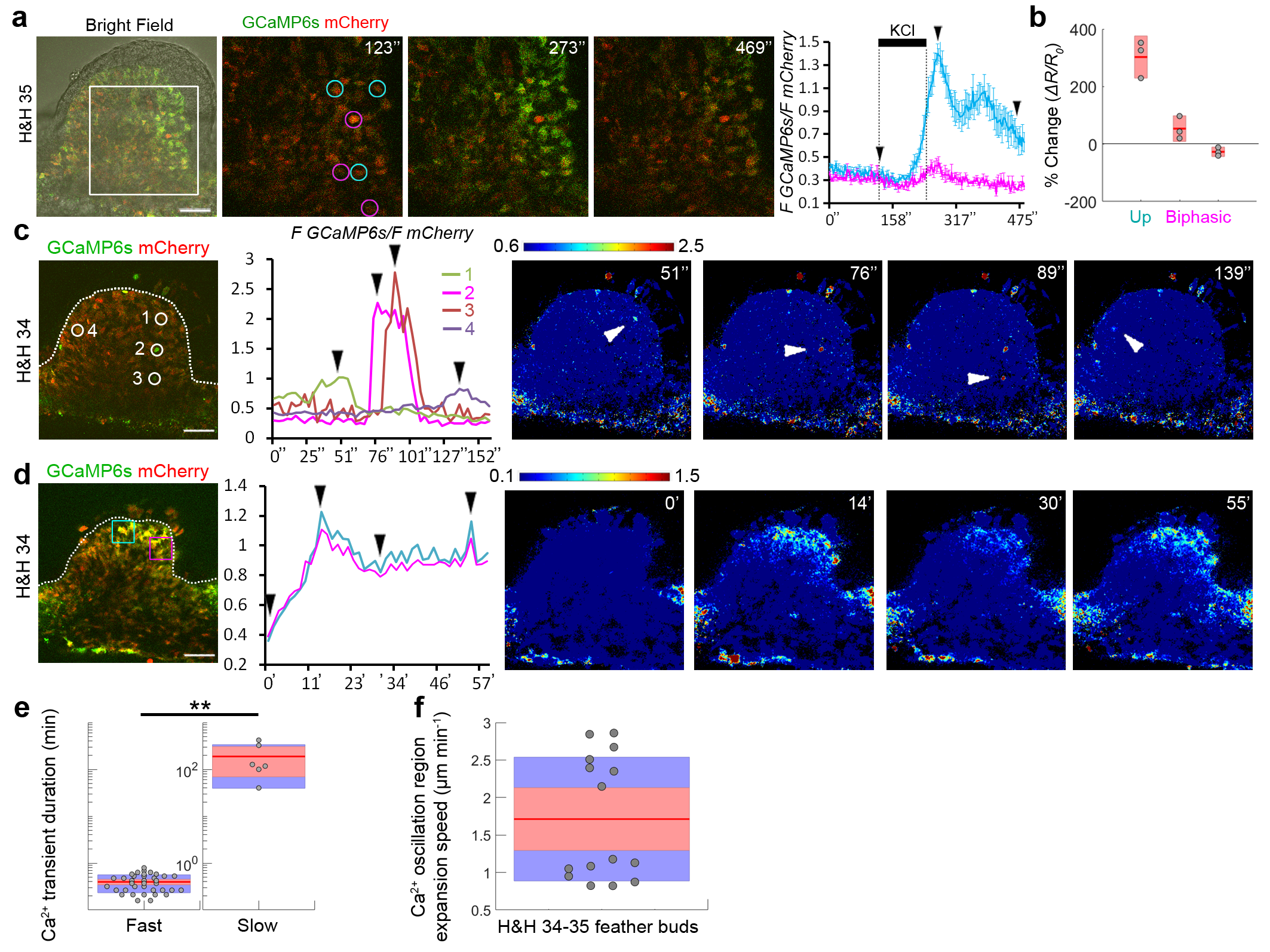


**Supplementary Figure 8 |** Heterogeneous patterns of KCl-induced and feather endogenous Ca^2+^ transients. (**a**) Representative recording showing heterogeneous mesenchymal KCl response in H&H 35 skin stripes. Enlarged images of the ROI (rectangle) are shown at three time points. Cyan circles denote cells with notable KCl-induced Ca^2+^ increases; Magenta circles denote cells with biphasic responses. Averaged *F GCaMP6s/F mCherry* ratio in these cells (n = 3 for each group) are shown in the line plot. Arrowheads highlight the three time points. ’’ denotes seconds. Data are presented as Mean ± s.e.m. Scale bar, 50 μm. (**b**) Relative cytosolic Ca^2+^ changes in different responses (n =3 for each type). Customized boxplot: Mean (red) ± s.d. (pink), 95% confidence interval (violet). Dots denote individual data points. (**c**) Representative recording of fast Ca^2+^ transients in H&H 34 skin stripes. Circles denote the ROIs used for producing the *F GCaMP6s/F mCherry* ratio plot. Ratiometric images were shown at selected time points (arrowheads). ’’ denotes seconds. (**d**) Representative recording of slow Ca^2+^ oscillations in H&H 34 skin strips. Dotted line highlights the epithelial-mesenchymal boundary. ROIs are established at anterior (cyan rectangle) and posterior (magenta rectangle) feather mesenchyme to plot *F GCaMP6s/F mCherry* ratio. Ratiometric images were shown at selected times (arrowheads). ’ denotes minutes. Scale bar, 50 μm. (**e**) Duration of fast (n = 36) and slow (n = 5) Ca^2+^ transients measured in skin stripes and explants at H&H 34-35. ** *P* < 0.01 (Wilcoxon Rank Test). (**f**) Expansion speed of the Ca^2+^ oscillation region in feather mesenchyme (n = 15).


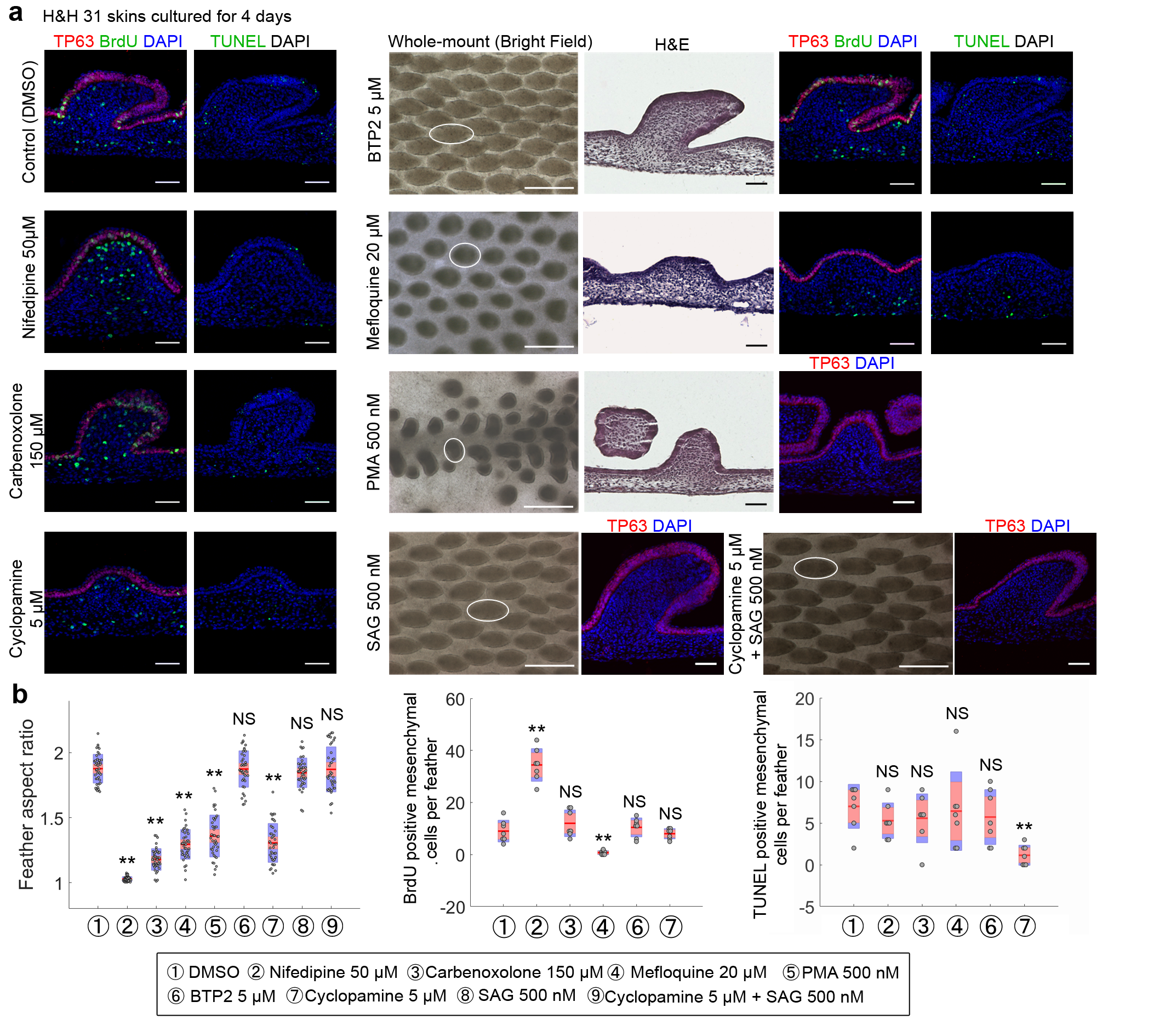


**Supplementary Figure 9 |** Cell proliferation and apoptosis in feather buds. (**a**) In control skins, feather buds became elongated filaments oriented along the original body anterior-posterior (A-P) axis. Mesenchymal cell proliferation (2 hr BrdU labeling) and apoptosis (TUNEL) occurred sparsely (n = 10/10). Nifedipine -treated skins had shorter feather buds without apparent A-P polarity while cell proliferation increased (n = 8/8). Carbenoxolone inhibited feather elongation and caused abnormal feather polarity without significantly affecting mesenchymal cell proliferation and apoptosis (n = 8/8). Cyclopamine-treated skins had extremely short feather buds without significant impact on mesenchymal cell proliferation. Meanwhile cell apoptosis decreased (n = 8/8). The phenotype was rescued by SAG in the presence of Cyclopamine (n = 4/4). SAG alone caused no discernable changes of feather morphology (n = 4/4). BTP2 did not produce notable changes of feather morphology (n = 8/8). Mefloquine inhibited feather elongation and mesenchymal cell proliferation. PMA inhibited feather elongation and caused abnormal feather orientation. Ellipses highlight changes of feather aspect ratio. Scale bars, 500 μm (whole-mount skin), 50 μm (section). (**b**) Quantification of feather bud aspect ratio (n = 40), proliferation and apoptosis in feather mesenchyme (n = 8). Customized boxplot: Mean (red) ± s.d. (pink), 95% confidence interval (violet). Dots denote individual data points. ** *P* < 0.01. NS, not significant (Wilcoxon Rank Test). H&E: Hematoxylin & Eosin staining. TP63 staining highlights feather epithelium. DAPI staining highlights nuclei.


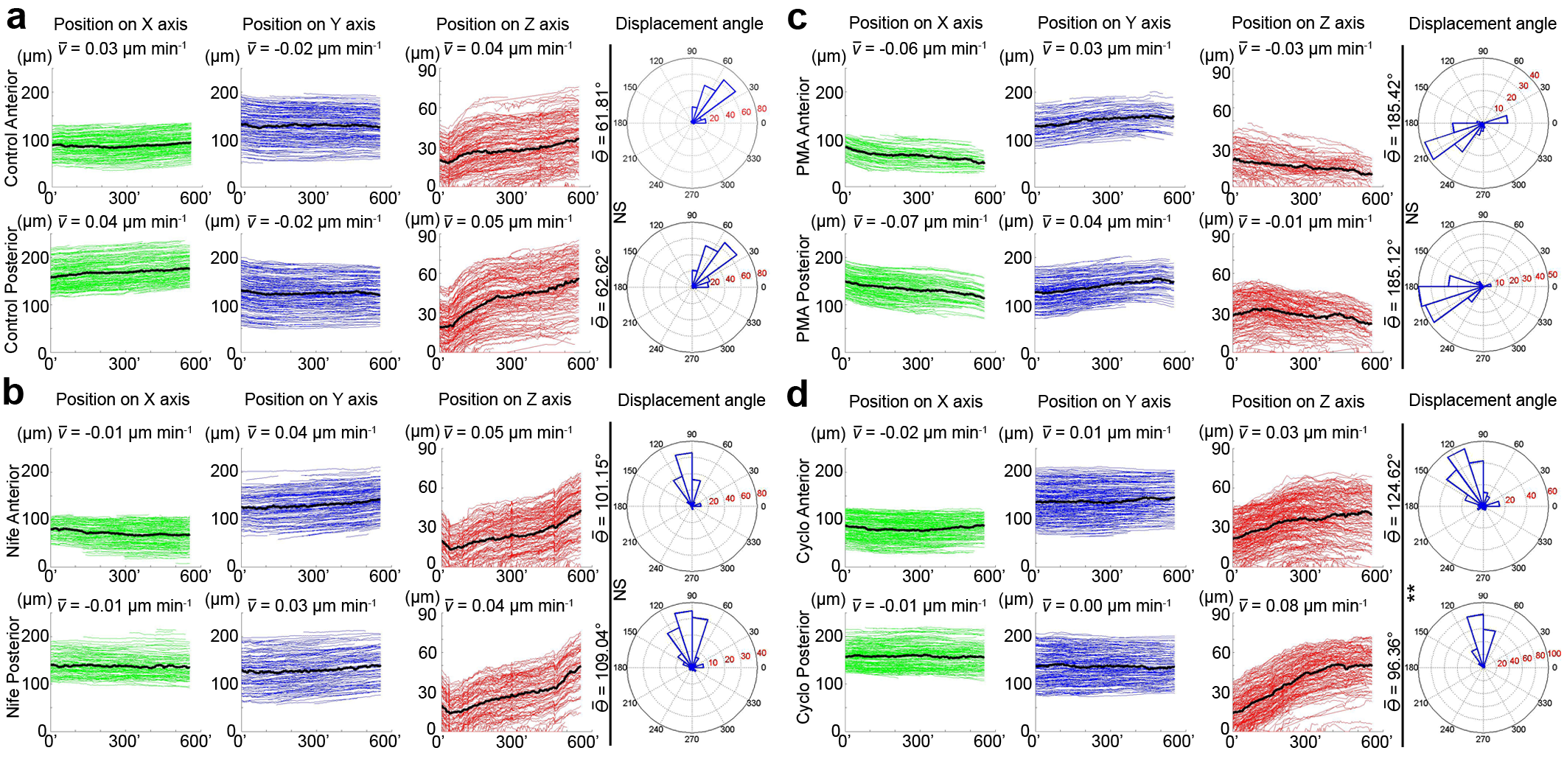


**Supplementary Figure 10 |** Differences of cell movement patterns in anterior and posterior halves of feather mesenchyme upon different treatments. (**a**) Plotting cell position along the X, Y, and Z axes over time in the anterior and posterior mesenchyme of control feather buds. ‘ in X axis denotes minutes. Black line is the averaged cell position over time. The averaged velocities are shown above the plots. Angle histogram plots illustrate the directions of cell displacement and the mean displacement angles are underlined. Both anterior (n = 180) and posterior (n = 198) halves of feather mesenchyme cells moved upward and posteriorly. The posterior population moved faster than the anterior counterpart. (**b**) In Nifedipine-treated skins, both anterior (n = 177) and posterior (n = 139) populations of feather mesenchymal cells moved upward but not posteriorly. (**c**) In PMA-treated skins, both anterior (n = 117) and posterior (n = 161) populations of mesenchymal cells moved downward and anteriorly. (**d**) In Cyclopamine-treated skins, the anterior population (n = 237) moved upward and anteriorly. The posterior population (n = 193) mainly moved upward. Their averaged displacement angles had statistically significant differences. ** *P* < 0.01. NS, not significant (Watson’s U^2^ test).


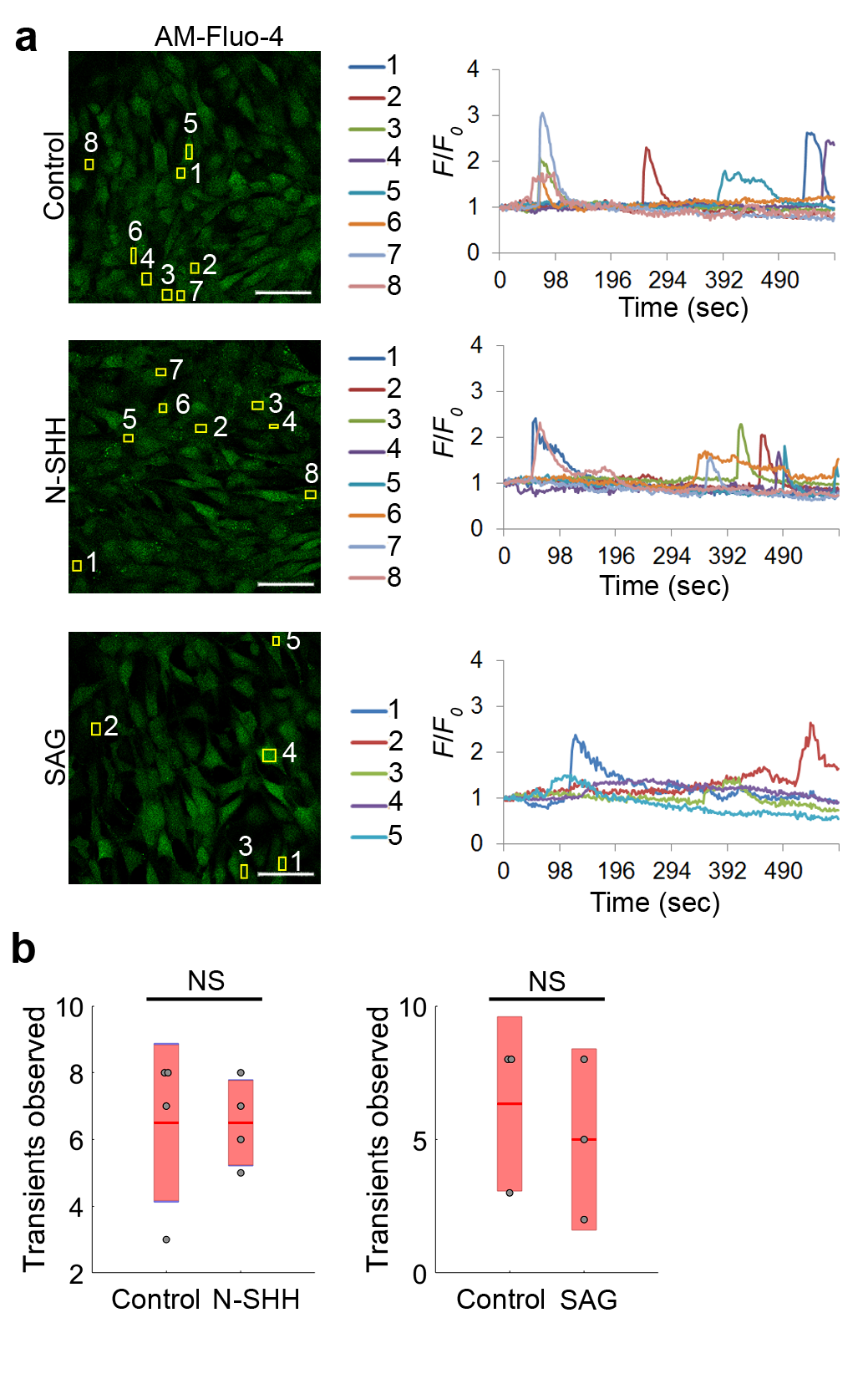


**Supplementary Figure 11 |** Activating SHH signaling did not significantly increase Ca^2+^ transients in cultured H&H 34 mesenchyme cells. (**a**) Cultured H&H 34 skin mesenchyme cells were labeled with AM-Fluo-4 Ca^2+^ indicator for 30 min and then pretreated with HBSS (control), 0.25 μM SHH N-terminal peptide, or 500 nM SAG for 20 min before imaging. ROI analysis was done on the spiking cells (yellow rectangles) to characterize the timing and fold of Ca^2+^ level changes. *F* represents the Fluo-4 fluorescence intensity averaged in the ROI and *F_0_* is the averaged Fluo-4 intensity from the first 4 seconds of the recording. Scale bar, 50 µm. (**b**) The spontaneous Ca^2+^ transients observed during the 10 min recording did not show significant differences between the recordings obtained in HBSS (Control, n = 4 recordings), SHH N-terminal peptide (n = 4), or SAG treated cells (n = 3). Customized boxplot: Mean (red) ± s.d. (pink). Dots denote individual data points. 95% confidence interval (violet). NS, not significant (Wilcoxon rank test).


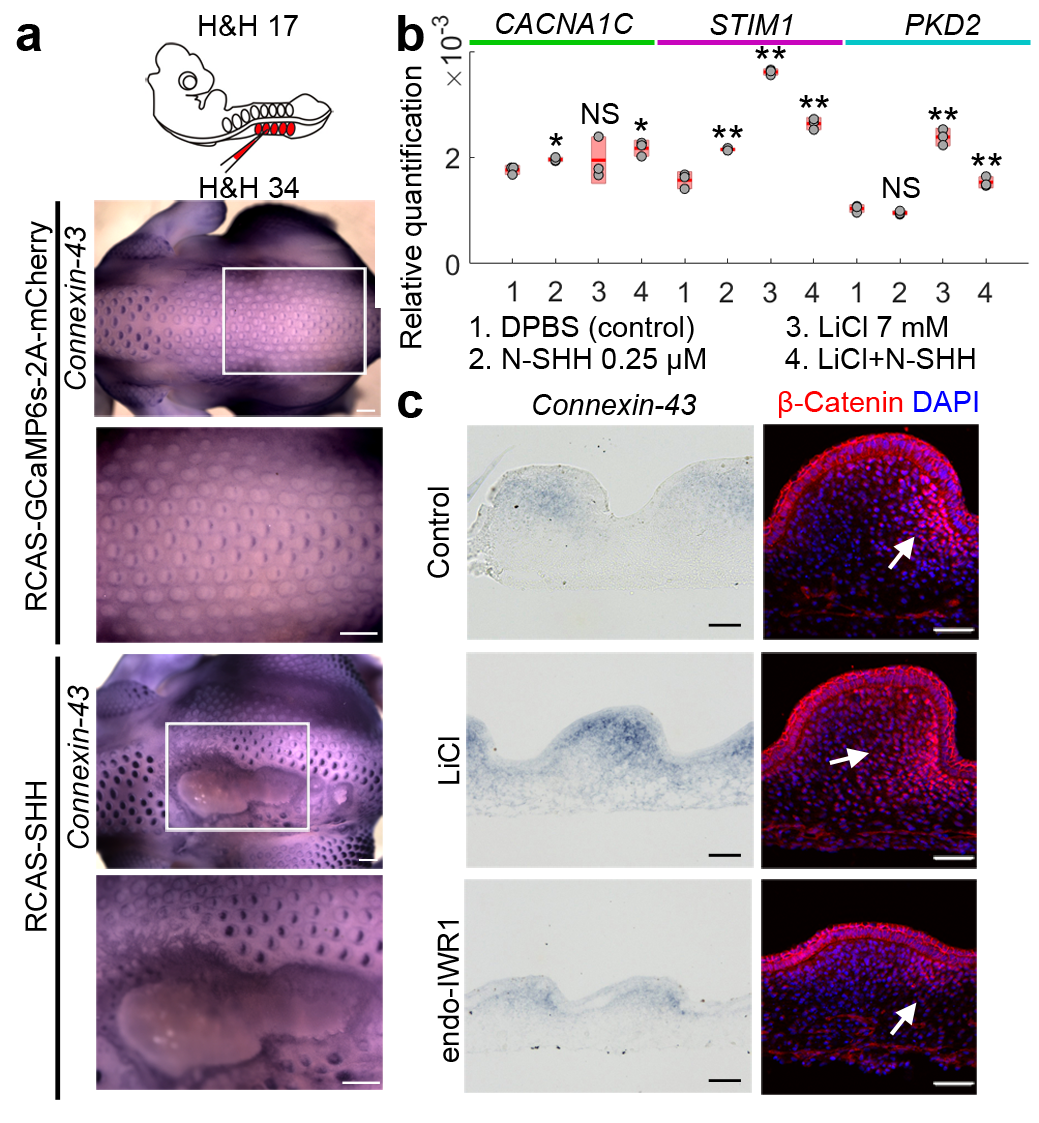


**Supplementary Figure 12 |** Influence of WNT and SHH signaling on the expression of Ca^2+^ related channels. (**a**) Injection of RCAS-GFP virus in the left half of the embryo produced no discernable changes of feather morphology or *Connexin-43* expression (n = 10/10). RCAS-SHH injection caused severe defects in skin and muscle development (n = 6/6), making it hard to evaluate its impact on feather development or *Connexin-43* expression. White rectangles highlight magnified regions. Scale bar, 500 µm. (**b**) qPCR results of Ca^2+^ related channels after 24 hr treatment with 0.25 µM mouse SHH N-terminal peptide, 7 mM LiCl (activates WNT signaling), or both reagents. With both SHH protein and LiCl present, expression of *STIM1* and *PKD2* were both upregulated. However, the fold change is smaller than SHH or LiCl alone. Customized boxplot: Mean (red) ± s.d. (pink), 95% confidence interval (violet). Dots denote individual data points. * *P* < 0.05, ** *P* < 0.01. NS, not significant (n = 3, two-sample Student’s t test). (**c**) Treating explant cultures from H&H 31 skins for 48 hrs with 7 mM LiCl elevated *Connexin-43* expression and the number of nuclear β-Catenin positive mesenchymal cells (arrows), while 10 μM endo-IWR1 (a WNT/β-Catenin signaling antagonist) treatment had the opposite effect (n = 4/4).

**Supplementary Table 1 |** Mesenchymal cell movement parameters in 4D imaging.

|  | Control | Control Anterior | Control Posterior | Nifedipine | Nifedipine Anterior | Nifedipine Posterior | PMA | PMA Anterior | PMA Posterior | Cyclopamine | Cyclopamine Anterior | Cyclopamine Posterior | Carbenoxolone |
| --- | --- | --- | --- | --- | --- | --- | --- | --- | --- | --- | --- | --- | --- |
| Total number of cells tracked | 378 | 180 | 198 | 316 | 177 | 139 | 278 | 117 | 161 | 430 | 237 | 193 | 427 |
| Averaged track length (µm) | 61.23 | 60.07 | 62.28 | 68.78 | 65.47 | 73 | 57.39 | 52.42 | 61.01 | 64.34 | 60.3 | 69.3 | 74.14 |
| Averaged track straightness | 0.39 | 0.34 | 0.44 | 0.36 | 0.4 | 0.32 | 0.5 | 0.48 | 0.51 | 0.36 | 0.29 | 0.45 | 0.47 |
| Averaged cell displacement | 21.04 | 18.03 | 23.79 | 21.12 | 21.32 | 20.86 | 24.44 | 21.02 | 26.93 | 20.31 | 14.02 | 28.03 | 33.5 |
| Averaged cell velocity* (µm min^-1^) | 0.07 | 0.05 | 0.08 | 0.07 | 0.08 | 0.07 | 0.09 | 0.09 | 0.1 | 0.07 | 0.05 | 0.09 | 0.1 |
| Averaged cell speed* (µm min^-1^) | 0.18 | 0.17 | 0.2 | 0.22 | 0.22 | 0.22 | 0.2 | 0.2 | 0.2 | 0.19 | 0.18 | 0.2 | 0.19 |

*Velocity is displacement divided by time. Speed is track length divided by time.

**Supplementary Table 2 |** Primers used for subcloning, *in situ* hybridization and RT-qPCR

| Gene | Application | Forward primer sequence | Reverse primer sequence |
| --- | --- | --- | --- |
| *GCaMP6s* | Subcloning | tatattgcggccgcatgggttctcatcatcatcatc | tatattactagtacacttcgctgtcatcatttgta |
| *GBS-GFP* | Subcloning | tggtatcgataagcttgattcgagga | ttttatcgatggccgctctagaactagtgga |
| *cSTIM1* | Subcloning | tttaagcttgaatcacactgcagctgggc | tctcgagttaagacacgatctcctcatccag |
| *mCherry-LOV-cSTIM1* | Subcloning  (BP reaction) | ggggacaagtttgtacaaaaaagcaggcttcaccatggcacaccatcaccaccatcac | ggggaccactttgtacaagaaagctgggtcttaagacacgatctcctcatccag |
| *shRNA-Connexin-43* | Subcloning | tgctgatacagtggtacattttcaagagaaatgtaccactgtatcagcttttttg | gttacaaaaaagctgatacagtggtacatttctcttgaaaatgtaccactgtatcagca |
| *CACNA1C* | *In situ* | agacagaaaaaccagggcgt | ctaatacgactcactatagggttgtctgctgcgttttcca |
| *CACNA1G* | *In situ* | caccactcatcatccacacc | ctaatacgactcactatagggagaatcagacggaacctggaatg |
| *CACNA1H* | *In situ* | tgcaagagtcacgaggattg | ctaatacgactcactatagggccgaacccatctgtagtgt |
| *CACNA2D2* | *In situ* | atcaatttccgtgagccggt | ctaatacgactcactatagggttgcttgccagaaccttg |
| *CACNG3* | *In situ* | ttcctgaggatgcggactac | gtaatacgactcactatagggagatgtcagtggaggggatg |
| *Connexin-43* | *In situ* | agagctcaaggtggtccaga | ctaatacgactcactatagggcatatttgggggaaccacag |
| *GJB3* | *In situ* | gctcagtggggtcaacaaat | ctaatacgactcactatagggagacagtgaggacgatgcaga |
| *GJC1* | *In situ* | ggtgctgatcgtctttcgga | ctaatacgactcactatagggagaacacatacagctcaccccg |
| *ITPR1* | *In situ* | catatgccggctctgctaca | ctaatacgactcactatagggagacgctctcaggtcatacgg |
| *ITPR2* | *In situ* | ggagcctgcgcaaaagaaaa | ctaatacgactcactatagggagacatggctctcatccgagg |
| *KCNMA1* | *In situ* | ttcgctgcaggacaaggaat | ctaatacgactcactatagggagactgagcaactcgacacctg |
| *ORAI1* | *In situ* | ggtggccatggtagaagttca | ctaatacgactcactatagggtaacagctggggagatg |
| *ORAI2* | *In situ* | gatccctccactcctgcttg | ctaatacgactcactatagggagatggtggtggtgtcgttct |
| *PANX1* | *In situ* | aatacccgattgtggagcag | ctaatacgactcactatagggcaaacttctcggacacagca |
| *PANX2* | *In situ* | ttatgcactgctggcttttg | gtaatacgactcactatagggcctgcaataatcctctgga |
| *PKD2* | *In situ* | ttgggcttcggaatggaaca | ctaatacgactcactatagggagatctttgacgcagcgagaca |
| *SHH* | *In situ* | ggaattcccag(ca)gitg(ct)aa(ag)ga(ag)(ca)(ag)i(gct)tia | tcattiatggaccca(ga)tc(ga)aaiccigc(tc)tc |
| *STIM1* | *In situ* | ccaggttagcggtgaacaat | ctaatacgactcactatagggtgattccagctccttctcg |
| *STIM2* | *In situ* | tgagttgacaacctgcttgc | ctaatacgactcactatagggagaaactttgcttgcagggcta |
| *TRPC1* | *In situ* | tggatgtggcacctgtcatc | ctaatacgactcactatagggagactggccagaaaatgccaacc |
| *TRPV2* | *In situ* | cagtgcagtgtcaaggggta | ctaatacgactcactatagggagacacacctgccttcaggattt |
| *Connexin-43* | RT-qPCR | agtgccctgggaaaacttct | ccaatagcaggattcggaaa |
| *ACTB* | RT-qPCR | gctatgaactccctgatggtc | ggactccatacccaagaaaga |
| *CACNA1C* | RT-qPCR | gctgttcttcatctacgccg | tctggaagttgttgttgcgg |
| *LEF1* | RT-qPCR | atccccagaacatccaacaa | ggaaaagtgctcatcgctgt |
| *PTCH1* | RT-qPCR | gagcagtacattgggcttcg | ctgtccaggggtttaggagg |
| *STIM1* | RT-qPCR | ggtgcagtggctcattacct | gcattgttcaccgctaacct |
| *PKD2* | RT-qPCR | atgagccatcgtctgggaac | tcaatgccatctgactgcga |
