# Supplementary material for: Calcium oscillations coordinate feather mesenchymal cell movement by SHH dependent modulation of gap junction networks
Source: Nat Commun. 2018 Dec 18;9:5377. doi: 10.1038/s41467-018-07661-5 (PMC6299091; doi:10.1038/s41467-018-07661-5)
Supplement: Supplementary file 41 — Description of Additional Supplementary Files [file 41467_2018_7661_MOESM41_ESM.pdf]

Supplementary Data 1. RPKM of genes encoding ion channels investigated in RNA-Seq analysis. Blue: Voltage-gated Ca^{2+} channels; Red: Ca^{2+} release activated channels; Purple: Ryanodine Receptors; Cyan: IP3 receptors; Orange: Transient receptor potential channels; Magenta: Gap junctions; Light green: Purinergic receptors; Brown: Ca^{2+} activated K^+ channels; Dark green: Na^+ channels.

Supplementary Movie 1 H&H 31 feather buds (skin stripe configuration) infected with RCAS-GCaMP6s-2A-mCherry exhibited KCl-induced Ca^{2+} influx in posterior mesenchyme. Green fluorescence is from GCaMP6s and red is from mCherry. The ratios of GCaMP6s to mCherry were calculated to generate ratiometric pseudocolor movie. See Fig. 2b for the color scale of the ratios.

Supplementary Movie 2 H&H 34 feather buds (skin stripe configuration) infected with RCAS-GCaMP6s-2A-mCherry exhibited KCl-induced Ca^{2+} influx in posterior mesenchyme. Green fluorescence is from GCaMP6s and red is from mCherry. The ratios of GCaMP6s to mCherry were calculated to generate ratiometric pseudocolor movie. See Fig. 2c. The color scale of the ratios is the same as shown in Fig. 2b.

Supplementary Movie 3 H&H 35 feather buds (skin stripe configuration) infected with RCAS-GCaMP6s-2A-mCherry exhibited KCl-induced Ca^{2+} influx along a bud. Green fluorescence is from GCaMP6s and red is from mCherry. The ratios of GCaMP6s to mCherry were calculated to generate ratiometric pseudocolor movie. See Fig. 2d. The color scale of the ratios is the same as shown in Fig. 2b.

Supplementary Movie 4 H&H 34 feather buds (skin explant configuration) infected with RCAS-GCaMP6s-2A-mCherry exhibited KCl-induced Ca^{2+} influx in mesenchyme. Green fluorescence is from GCaMP6s and red is from mCherry. The ratios of GCaMP6s to mCherry were calculated to generate ratiometric pseudocolor movie. See Supplementary Fig. 4a. The color scale of the ratios is the same as shown in Fig. 2b.

Supplementary Movie 5 H&H 35 feather buds (skin explant configuration) infected with RCAS-GCaMP6s-2A-mCherry exhibited more robust KCl-induced Ca^{2+} influx in mesenchyme. Green fluorescence is from GCaMP6s and red is from mCherry. The ratios of GCaMP6s to mCherry were calculated to generate a ratiometric pseudocolor movie. See Fig. 2f. The color scale of the ratios is the same as shown in Fig. 2b.

Supplementary Movie 6 Pretreating H&H 35 feather buds (skin explant configuration) with the VGCC blocker, Nifedpine, significantly reduced KCl-induced Ca^{2+} influx. Nifedipine was administered from 0 sec to 360 sec. KCl was administered from 180 sec to 360 sec. See Fig. 2g. The color scale of the ratios is the same as shown in Fig. 2b.

Supplementary Movie 7 Heterogeneity of KCl response in dissociated H&H 35 skin mesenchymal cells. Mesenchymal cells were cultured and loaded with AM-Fura-2. The ratios of Fura-2 fluorescence intensity at 350 nm excitation wavelength (F350) over F380 were calculated as readouts of Ca^{2+} levels, and used for making the pseudocolor images. See Fig. 3a for the color scale of the ratios.

Supplementary Movie 8 Pretreating H&H 35 feather buds (skin explant configuration) with the gap junction blocker, Carbenoxolone, significantly reduced KCl-induced Ca^{2+} influx. The skins were pretreated for 30 min with 150 µM Carbenoxolone before the administration of KCl. See Fig. 2h. The color scale of the ratios is the same as shown in Fig. 2b.

Supplementary Movie 9 Pretreating H&H 35 feather buds (skin explant configuration) with the gap junction blocker, PMA, significantly reduced KCl-induced Ca^{2+} influx. The skins were pretreated for 30 min with 500 nM PMA before the administration of KCl. See Supplementary Fig. 6c.

Supplementary Movie 10 CRAC channel mediated Ca^{2+} influx was seen in H&H 35 feather buds pretreated with 0 mM Ca^{2+} solution including Thapsigargin followed by 2 mM Ca^{2+} solution. 0 mM Ca^{2+} solution including Thapsigargin was administered for 30 min before the application of 2 mM Ca^{2+} solution. This will deplete the ER Ca^{2+} store and hence activate CRAC channels if there are any. Hence the Ca^{2+} influx after applying Ca^{2+} containing solution validates the presence of active CRAC channels in feathers. See Fig. 2i. The color scale of the ratios is the same as shown in Fig. 2b.

Supplementary Movie 11 BTP2 (CRAC channel blocker) largely reduced Ca^{2+} influx induced by depletion of ER Ca^{2+} store. 5 µM BTP2 was added to the pretreatment solution and the Ca^{2+} containing solution. See Fig. 2j. The color scale of the ratios is the same as shown in Fig. 2b.

Supplementary Movie 12 Suppression of KCl-induced Ca^{2+} influx by the VGCC blocker, Nitrendipine. Pretreatment of cultured mesenchymal cells with 10 µM Nitrendipine largely dampened the KCl-induced Ca^{2+} influx. The further decrease of cytoplasmic Ca^{2+} may be due to depolarization-induced inhibition of CRAC channel conductance. See Fig. 3c for the color scale of the ratio.

Supplementary Movie 13 10 µM La^{3+} (a CRAC channel inhibitor) treatment significantly decreased cytoplasmic free Ca^{2+} levels in dissociated H&H 35 skin mesenchymal cells. Cells exhibiting KCl-induced Ca^{2+} influx also demonstrated La^{3+}-induced decreases in cytoplasmic free Ca^{2+} concentrations, implying the presence of both VGCCs and CRAC channels. See Fig. 3c for the color scale of the ratio.

Supplementary Movie 14 H&H 35 feather buds (skin stripe configuration) infected with RCAS-GCaMP6s-2A-mCherry exhibited KCl-induced Ca^{2+} influx in distal mesenchyme. Green fluorescence is from GCaMP6s and red is from mCherry. The ratios of GCaMP6s to mCherry were calculated to generate ratiometric pseudocolor movie. See Supplementary Fig. 8a. Color scale: Blue: 0.25, Red: 0.45.

Supplementary Movie 15 Light-induced Ca^{2+} influx in HeLa cells infected with lentivirus encoding mCherry-LOV-cSTIM1 (opto-cCRAC). jRCaMP1b reported change of Ca^{2+} level. See Fig. 4a.

Supplementary Movie 16 Time-lapse Ca^{2+} imaging in H&H 35 mesenchymal cells infected with RCAS virus encoding opto-cCRAC. AM-Fluo-4 reported change of Ca^{2+} level. See Fig. 4b.

Supplementary Movie 17 Ratiometric Ca^{2+} imaging of elongating feather buds from a H&H 34 skin stripe showing fast, sporadic Ca^{2+} transients. See Supplementary Fig. 8c for the color scale of the ratio.

Supplementary Movie 18 Ratiometric Ca^{2+} imaging of elongating feather buds from a H&H 34 skin stripe showing slow Ca^{2+} transients in posterior-distal mesenchyme. See Supplementary Fig. 8d for the color scale of the ratio.

Supplementary Movie 19 4D ratiometric Ca^{2+} imaging of elongating feather buds from H&H 34 skins over 9 hrs. A zone of synchronized oscillations emerged initially at the posterior-distal mesenchyme and gradually expanded in area. See Fig. 5a for the color scale of the ratio.

Supplementary Movie 20 An XY plane virtual section (18 µM thick) of the recording in a H&H 34 control skin explant. See Fig. 5a for the color scale of the ratio.

Supplementary Movie 21 An XZ plane virtual section (18 µM thick) of the recording in a H&H 34 control skin explant. See Fig. 5a for the color scale of the ratio.

Supplementary Movie 22 4D ratiometric Ca^{2+} imaging of elongating feather buds from a H&H 34 skin treated with the VGCC blocker, Nifedipine. Only sparse Ca^{2+} transients were observed. See Fig. 5a for the color scale of the ratio.

Supplementary Movie 23 An XY plane virtual section (18 µM thick) of the recording in a H&H 34 skin treated with the VGCC blocker, Nifedipine. See Fig. 5a for the color scale of the ratio.

Supplementary Movie 24 An XZ plane virtual section (18 µM thick) of the recording in a H&H 34 skin treated with the VGCC blocker, Nifedipine. See Fig. 5a for the color scale of the ratio.

Supplementary Movie 25 4D ratiometric Ca^{2+} imaging of elongating feather buds from a H&H 34 skin treated with the gap junction blocker, PMA. Only sparse Ca^{2+} transients were observed. See Supplementary Fig. 6d for the color scale of the ratio.

Supplementary Movie 26 An XY plane virtual section (18 µM thick) of the recording in a H&H 34 skin treated with the gap junction blocker, PMA. See Supplementary Fig. 6d for the color scale of the ratio.

Supplementary Movie 27 An XZ plane virtual section (18 µM thick) of the recording in a H&H 34 skin treated with the gap junction blocker, PMA. See Supplementary Fig. 6d for the color scale of the ratio.

Supplementary Movie 28 4D cell nucleus imaging of elongating feather buds from a H&H 34 skin and tracking of mesenchymal cell positions. Skins were infected with RCAS virus encoding H2B-GFP to highlight the cell nuclei. Most cells moved in the posterior-upward directions. See Fig. 6 for cell tracking results.

Supplementary Movie 29 4D cell nuclear imaging of elongating feather buds treated with Nifedipine and tracking of mesenchymal cell positions. Most cells moved upward without a notable bias in the anterior or posterior direction. See Fig. 6 for cell tracking results.

Supplementary Movie 30 4D cell nuclear imaging of elongating feather buds treated with Carbenoxolone and tracking of mesenchymal cell positions. Most cells moved upward without a notable bias in the anterior or posterior direction. See Fig. 6 for cell tracking results.

Supplementary Movie 31 4D cell nuclear imaging of elongating feather buds treated with PMA and tracking of mesenchymal cell positions. Most cells moved anteriorly and downward. See Supplementary Fig. 6e,f for cell tracking results.

Supplementary Movie 32 4D ratiometric Ca^{2+} imaging of elongating feather buds from a H&H 34 skin treated with the SHH signaling inhibitor, Cyclopamine. Mosaic Ca^{2+} transients were observed but no synchronized oscillations. See Fig. 7d for the color scale of the ratio.

Supplementary Movie 33 An XY plane virtual section (18 µM thick) of the recording in a H&H 34 skin treated with the SHH signaling inhibitor, Cyclopamine. See Fig. 7d for the color scale of the ratio.

Supplementary Movie 34 An XZ plane virtual section (18 µM thick) of the recording in a recording from H&H 34 skins treated with the SHH signaling inhibitor, Cyclopamine. See Fig. 7d for the color scale of the ratio.

Supplementary Movie 35 4D cell nuclear imaging of elongating feather buds treated with Cyclopamine and tracking of mesenchymal cell positions. Most cells moved upward and then moved downward at the end of the recording. Anterior mesenchymal cells moved more anteriorly. See Fig. 7e,f for cell tracking results.

Supplementary Movie 36 Applying the SHH N-terminus protein or SHH agonist SAG did not significantly increase spontaneous Ca^{2+} transients in dissociated H&H 34 mesenchymal cells. Cells were loaded with the AM-Fluo-4 Ca^{2+} indicator for 30 min and pretreated with HBSS (control), N-SHH or SAG for 20 min before the recordings. See Supplementary Fig. 11 for quantification results.

Supplementary Movie 37 Pretreating feather buds with Cyclopamine did not significantly reduce KCl-induced Ca^{2+} influx. The skins were pretreated for 3 min with 5 µM Cyclopamine before the administration of KCl. See Supplementary Fig. 4b for the color scale of the ratio.

Supplementary Movie 38 Pretreating feather buds with SAG did not significantly reduce KCl-induced Ca^{2+} influx. The skins were pretreated for 3 min with 1 µM SAG before the administration of KCl. See Supplementary Fig. 4d for the color scale of the ratio.
